# Integrating the effects of driving forces on ecosystem services into ecological management: A case study from Sichuan Province, China

**DOI:** 10.1371/journal.pone.0270365

**Published:** 2022-06-23

**Authors:** Ying Huang, Tian Feng, Shaofei Niu, Desheng Hao, Xiaoyu Gan, Bo Zhou

**Affiliations:** 1 College of Architecture and Environment, Sichuan University, Chengdu, 610064, PR China; 2 School of Public Administration, Sichuan University, Chengdu, 610064, PR China; Đại Học Duy Tân: Dai Hoc Duy Tan, VIET NAM

## Abstract

Driving forces are the factors that lead to the observed changes in the quantity and quality of ecosystem services (ESs). The relationship between driving forces and ESs involves considerable scale-related information. Place-based ecological management requires this information to support local sustainable development. Despite the importance of scale in ES research, most studies have only examined the association between ESs and their drivers at a single level, and few studies have examined this relationship at various scales or analyzed spatial heterogeneity. The purpose of this paper is to explore the significance of the scale-dependent effects of drivers on ESs for localized ecological management. The biophysical values of ESs were calculated using several ecological simulation models. The effects of driving forces on ESs were explored using the geographically weighted regression (GWR) model. Variations in the effects of driving forces on ESs were examined at three scales: provincial, ecoregional, and subecoregional scales. Finally, canonical correlation analysis was used to identify the major environmental factors associated with these variations in each ecoregion. Our results show that (1) the distribution of soil conservation and water yield is highly heterogeneous; (2) four driving forces have significant positive and negative impacts on soil conservation and water yield, and their effects on the two services vary spatially (p < 0.05); (3) the impacts of drivers on ESs vary across different spatial scales, with a corresponding shift in the related environmental factors; and (4) in the study area, at the provincial scale, physical, topographical, and biophysical factors were key factors associated with the variations in the relationship between ESs and drivers, and at the ecoregional and subecoregional scales, physical, socioeconomic, topographical, and biophysical factors all contributed to these changes. Our results suggest that significant differences in topographical conditions (e.g., altitude, slope) can be incorporated for exploring the relationship between drivers and ESs and optimizing ecological management at the provincial scale, whereas significant differences in physical and socioeconomic conditions (e.g., urbanization levels, human activity, vegetation coverage) are more meaningful for localized ecological management at the ecoregional and subecological scales. These findings provide a basis for understanding the relationship between drivers and ESs at multiple scales as well as guidelines for improving localized ecological management and achieving sustainable development.

## 1 Introduction

Ecosystem services (ESs) can be defined as the goods (e.g., food and timber) and services (e.g., climate regulation and carbon sequestration) humans obtain from ecosystems [1,2]. ESs are of great concern to the public and governments worldwide for their contributions to human well-being [3]. There is growing recognition that the ultimate goal of ES research is to guide decision-making to facilitate ecological sustainability [4–7].

ESs are dynamic and may change in space and time as a result of natural and anthropogenic driving forces [3,8–12]. In this study, driving forces were defined as factors leading to the observed quantitative and qualitative changes in ESs [13,14]. The main drivers that determine ES changes include the following types: climate change, land-use change, and socioeconomic factors [15–22]. As a global phenomenon, climate change is one of the most challenging issues of our time, with a tremendous impact on the provision of ESs by changing the biophysical structures and processes of the ecosystem [12,14,21,23]. Specifically, Asmus et al. [24] observed that climate change in coastal environments affects the physical aspects of oceans, such as temperature, water acidification, and the mean sea level, thereby degrading the ecological functions of ocean ecosystems and consequently restricting or removing the services they provide to society and nature. Land use and land cover changes (LULCC) have been identified as one of the main driving forces responsible for temporal and spatial variations in ESs and functions [13,18,23]. Several studies have shown that land use changes the quantity and quality of ESs by affecting the ecosystem composition and structure, transforming the ecological attributes of ecosystems, and altering biophysical and biochemical processes [7,8,18,25]. Macro-socioeconomic factors are often considered the key factors that reflect the level of socioeconomic development and intensity of human activities and lead to large-scale modifications in ESs [15,26]. For example, policies with different incentives have different impacts on the provision of ESs. Lu et al. [27] analyzed four key ecosystem services on the Loess Plateau under Chinese ecological rehabilitation policies and concluded that policies with economic incentives increased soil conservation and carbon sequestration while reducing water yield in certain climate conditions. Wang et al. [26] noted that governmental programs such as “Grain for Green” can improve the ES supply by increasing the proportion of forestland.

A considerable amount of scale-related information is also involved in the relationship between ESs and driving forces [4,19,28,29]. Some studies have shown, for example, that the impacts of land-use changes on ESs differ widely depending on the spatial scales [7,20]. Ahmed et al. [8] concluded that examining more local-scale drivers of forest biomass and water yield can help managers better understand the localized effects of climate change on ESs. These findings demonstrated that estimation of the relationships between ESs and their drivers at incorrect scales may lead to oversight problems in decision-making [30,31]. More specifically, Sun et al. [19] found that drivers explained less of the variation in ES supply at the metropolitan scale than at the state scale. At the state scale, ES supply was mainly affected by population density, road density, patch density, and average annual precipitation, while at the metropolitan scale, it was mainly affected by population density, patch density, and average annual precipitation.These results suggest that localized management based on the relationship between ES supply and road density at the metropolitan scale may not be able to coordinate this relationship to manage the service to produce desired outcomes at the state scale. In this context, understanding the effects of driving forces on ESs from a multiscale perspective, identifying factors related to them, and determining how they are related would help us to optimize scale-appropriate decisions on localized management and strengthen sustainable development [32,33].

There is increasing awareness of the importance of scale effects for ES analysis and valuation of ESs [34]. Scale effects usually refer to the differences in ecological patterns and processes due to the scale of observation and contain two dimensions, spatial and temporal [3,28,35]. Previous studies related to the scale effects of ES research have mainly focused on the following aspects: supply, demand, and measurement [3,10,31,36]. The scale effects of ES supply can be embodied in the spatial pattern of individual ES [3,5,31], ES interactions [37–41], and ecosystem service bundles (ESB) [3,31,42]. The ES demand by stakeholders at different spatial scales, such as municipal, provincial or national, and international levels, has its own emphasis. Specifically, local stakeholders may emphasize local heritage, culture, or facilities, while national and/or global stakeholders may pay greater attention to the conservation of nature and biodiversity [28]. The ES supply-demand relation is even more complex [43], and such a relation at different scales may result in a scale mismatch [19]. In addition, the scales of observation determine the ES output [42]. Specific scales, such as regular grids or administrative districts (e.g., the township scale and county scale), are widely applied as the research unit [3,5,6]. Despite recognizing the importance of scale effects in ES research, most studies have only examined the associations between ESs and their drivers at a single scale, such as regular grids or administrative districts [6], and few have examined these relationships at different scales [10] or analyzed spatial heterogeneity[7,31].

The analysis of scale dependency is especially important to the ecological management of Sichuan Province.

As an important ecological barrier in the upper reaches of the Yangtze River, Sichuan Province faces huge challenges, especially the issue of ecological sustainability [33,44]. Sichuan Province is characterized by significant regional heterogeneity, which is related to the complexity of geomorphic conditions such as mountains, plateaus, hills, basins, and plains and the diversity of climate zones, including the Plateau Climate Zone, North Subtropical Zone, and Middle Subtropical Zone [44]. Previous research has shown that the impacts of drivers on ESs vary widely across different spatial scales in this area. For instance, [45] found that climate and topography were the main factors affecting the spatial distribution of soil conservation at the provincial scale, while vegetation factors played a significant role at the local scale. Hence, to maintain the vital functions of ESs and promote sustainable development of Sichuan Province, it is necessary to examine the relationships between ESs and driving forces at multiple spatial scales and provide references for regional ecological management.

Through a case study of Sichuan Province, China, this study investigates the significance of the scale dependency of drivers on ESs for localized ecological management. The biophysical values of ESs were calculated and mapped in 2018. The geographically weighted regression (GWR) model was utilized to explore the effects of driving forces on ESs. Variations in their effects on ESs were examined at provincial, ecoregional, and subecoregional scales. Finally, canonical correlation analysis was used to identify the major factors associated with these variations at each spatial scale. The specific objectives of this study were to (1) analyze variations in the effects of driving forces on ESs at three spatial scales; (2) identify the main environmental factors associated with these variations at each spatial scale; and (3) discuss the implications of regional ecological management by analyzing the relationships between environmental factors and drivers.

## 2 Materials and methods

### 2.1 Study area

**Fig 1:**
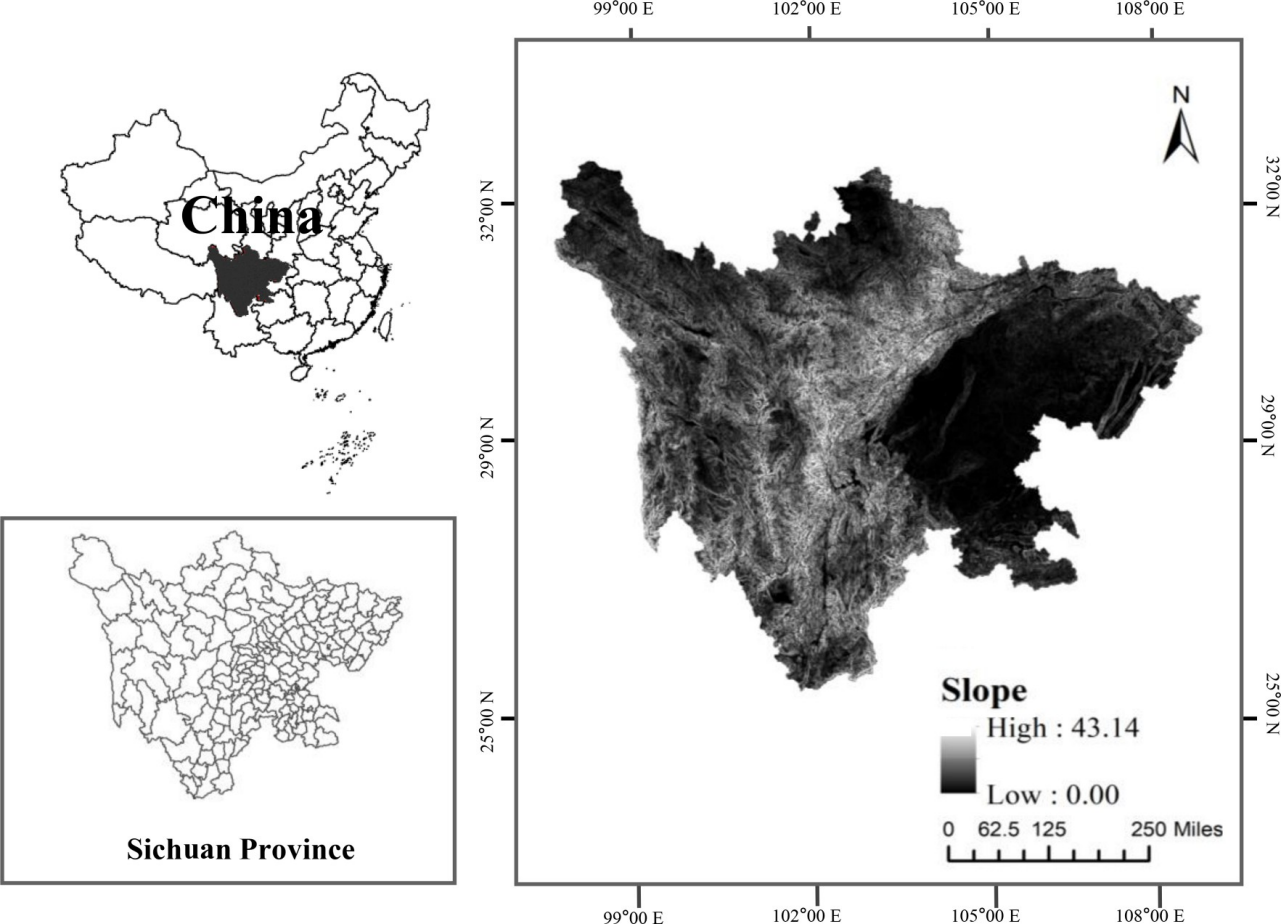
Location of the study area. (Reprinted from [Fig 1] under a CC BY license, with permission from [Resource and Environment Science and Data Center], original copyright [2018]).

Sichuan Province is located at 97°21’ E to 108°31’ E and 26°03’ N to 34°19 N, Southwest China, covering a total area of 4.86 × 10^5^ km^2^ [33] (Fig 1). The elevation of the study area ranges from 212 m to 6,904 m and gradually decreases from the western to the eastern side of the study area [46]. Sichuan Province is made up of plateaus and mountains with elevations exceeding 3,000 m in the west and basins and hills with elevations ranging from 500 to 2,000 m in the east [44]. There are significant differences in climate characteristics among different regions. The eastern area is characterized by a subtropical humid monsoon climate with an annual temperature of 16–18°C and annual precipitation of 900–1,200 mm. The western region is characterized by an alpine climate with an annual temperature of 4–12°C and annual rainfall of 500–900 mm [45]. In 2018, forestland accounted for 45.57% of the total study area, followed by grassland (25.13%), cropland (13.86%), other types of land (7.88%), built-up land (3.93%), water bodies (2.12%) and garden land (1.5%). The spatial distribution of the main land-use types showed significant regional differences [33]. Known as one of the most important industrial centers in western China, Sichuan Province is currently undergoing rapid urbanization [44]. In 2018, the total gross domestic product of this area reached 40,678.13 billion yuan, and the total population was approximately 83.41 million persons, accounting for 4.4% of the total gross domestic product of China and 5.98% of its national population [46].

### 2.2 Data sources

The data used in this study were obtained using the following sources.

(1) Land use/cover (LULC) data in 2018 with a spatial resolution of 30 m were retrieved from the Resource.

and Environment Science and Data Center (https://www.resdc.cn/data.aspx?DATAID=264). In this study, the land-use types were classified into six types, cropland, forestland, grassland, waterbody, built-up land, and bare land, based on the “Chinese Classification Criteria of land use” (GB/T21010-2007).

(2) Digital elevation model (DEM) data with a resolution of 90 m were obtained from the Resource and Environment Science and Data Center (https://www.resdc.cn/data.aspx?DATAID=284).
(3) The normalized difference vegetation index (NDVI) with a resolution of 1000 m was retrieved from the Resource and Environment Science and Data Center (https://www.resdc.cn/data.aspx?DATAID=257).
(4) Meteorological data from 42 meteorological stations within the study area were collected from the Resource and Environment Science and Data Center (https://www.resdc.cn/data.aspx?DATAID=230).
(5) Soil property data (topsoil sand, topsoil silt, topsoil clay, and topsoil carbon) with 1000 m spatial resolution were provided by the National Tibetan Plateau Data Center http://data.tpdc.ac.cn/zh-hans/data/611f7d50-b419-4d14-b4dd-4a944b141175/?q=%E5%9C%9F%E5%A3%A4%E6%95%B0%E6%8D%AE [47]. The soil classification is FAO-90 [40].

In this work, we referred to the environmental factors as spatial variables with high spatial heterogeneity. According to the environmental features and data availability of Sichuan Province, ten environmental factors in the economic, social, ecological, and environmental aspects were selected [46]. Table 1 gives a brief description of these environmental factors. The physical and topographical factors were calculated in ArcGIS 10.6 software (Esri, US) [48]. Socioeconomic factors were retrieved from the Resource and Environment Science and Data Center (https://www.resdc.cn/DOI/doi.aspx?DOIid=33 and https://www.resdc.cn/DOI/DOI.aspx?DOIid=32).

**Table 1:** Variable descriptions.

Variable category	Variable	Description
Physical factor	PBL	The proportion of built-up land
	PCL	The proportion of cropland
	PGL	The proportion of grassland
	PFL	The proportion of forestland
Socioeconomic factor	GDP	Gross domestic product
	POP	Population density
Topographical factor	DEM	Digital Elevation Model
	Slope	
	RDLS	The relief degree of the land surface
Biophysical factor	NDVI	Normalized Difference Vegetation Index

**Fig 2:**
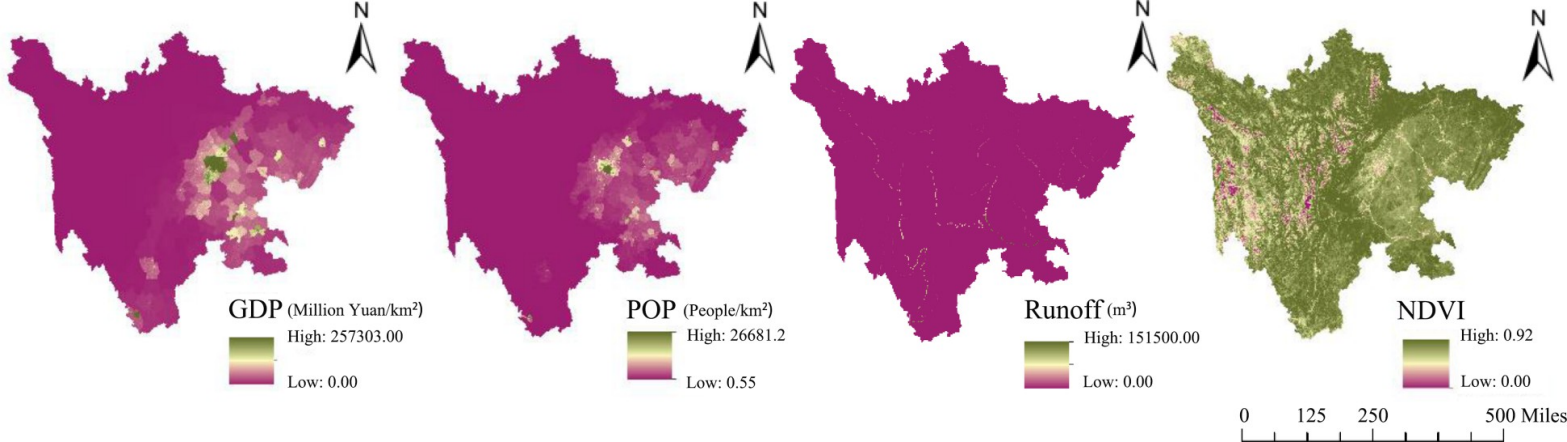
Spatial distribution of the four drivers in Sichuan Province.

In addition, the driving forces of ESs, including gross domestic product (GDP), population density (POP), runoff, and NDVI, were also selected from these environmental factors based on previous studies [26,49–51]. The spatial distributions of the four drivers are displayed in Fig 2. Finally, to unify the resolution of differences and improve the accuracy of the results, all the data were interpolated and resampled to 1000 m resolution by using ArcGIS 10.6 software (Esri, US).

### 2.3 Ecosystem service quantification

Choosing the appropriate indicators to quantify ESs is difficult because the main ESs differ from ecosystem to ecosystem. There is a need to select ESs that are most representative of the ecological problems facing the study area [51]. Sichuan Province has experienced long periods of soil erosion [45] and water shortage [52], which result in soil fertility decline, habitat loss, and local land degradation. Therefore, considering the main ecological challenges, strong linkage to the study area, and data availability, two ESs, namely, soil conservation (SC) and water yield (WY), were selected and estimated in this work.

#### Soil conservation

Soil conservation (SC) is a service that describes the ability of ecosystems to reduce soil erosion and mitigate the sedimentation of rivers, lakes, and reservoirs through the interception, absorption, infiltration, and fixation of root systems [53]. SC can be calculated as the difference between potential soil erosion and actual soil erosion [45,51]. In this study, the Revised Universal Soil Loss Equation (RUSLE) was used to estimate SC [54]. This equation is expressed as:

SC=R×K×LS×(1‐C×P) (1)

where SC represents the amount of annual SC (t ha^-1^); R is the rainfall erosivity index (MJ mm ha^-2^ ha^-1^), which is calculated by using Wischmeier`s monthly scale formula [55]; K is the soil erodibility index (t h MJ^-1^ mm^-1^), which is calculated by using an equation provided by the United States Department of Agriculture [56]; LS is the topographic factor (dimensionless); C is the vegetation cover factor (dimensionless, ranging from 0 to 1); and P is the conservation practice factor (dimensionless, ranging from 0 to 1).

$$R=\sum_{i=1}^{12}\left(1.735\times 10^{1.5\times \mathrm{lg}\frac{p_{i}{}^{2}}{P}-0.8188}\right)$$ (2)

$$\begin{array}{l} K=0.1317\times \left\{0.2+0.3EXP[-0.0256\times SAN\times (1-\frac{SIL}{100})]\right\}\times \\ {\left(\frac{SIL}{CLA+SIL}\right)}^{0.3}\times \left[1-\frac{0.25\times C}{C+EXP(3.72-2.95*C}\right]\times \\ \left[1-\frac{0.7\times SN_{1}}{SN_{1}+EXP(-5.51+22.9\times SN_{1})}\right],SN_{1}=1-\frac{SAN}{100} \end{array}$$ (3)

where P is the average annual precipitation (mm), P_i_ is the average monthly precipitation (mm), K is the soil erodibility index, and SAN, SIL, CLA, and C are the contents of sand, silt, clay, and organic carbon, respectively.

#### Water yield

Water yield (WY) is one of the most valuable services to society and an integral ecosystem component that regulates living biomass, the carbon cycle, and the energy budget [8]. WY is a measurement of the total amount of water that flows out of a drainage basin within a specified period of time [8] and is widely determined by the difference between precipitation and evapotranspiration [21,39]. In this work, the InVEST (Integrated Valuation of Ecosystem Services and Trade-offs) model, which is widely used for ES evaluation and mapping, was used to assess WY [57]. Its calculation formula is as follows:

$$Y_{x}=(1-\frac{AET_{x}}{P_{x}})\times P_{x}$$ (4)

$$\frac{AET_{x}}{P_{x}}=\frac{1+\omega_{x}R_{x}}{1+\omega_{x}R_{x}+(\frac{1}{R_{x}})}$$ (5)

$$R_{x}=\frac{K_{x}\times ET_{0}}{P_{x}}$$ (6)

$$\omega_{x}=Z\frac{AWC_{x}}{P_{x}}$$ (7)

where Y_X_ is the annual water yield (mm), P_x_ is the annual precipitation (mm m^-1^), AET is the actual evapotranspiration (mm m^-1^), **ω**_**x**_ is a dimensionless ratio of plant-accessible water storage to precipitation, R_x_ is the aridity index for certain land use/cover types, K_x_ refers to the vegetation evapotranspiration coefficient associated with land-use/cover types, ET_0_ represents the potential evapotranspiration calculated by the algorithm of the FAO [58], z is a hydrogeological constant that is computed by the method of [59] and AWC is the volumetric plant available water content.

### 2.4 Driver effect analysis

In this study, the geographically weighted regression (GWR) model was adopted to identify the spatially nonstationary relationships between ESs and driving forces. The GWR model is a local linear regression method that extends ordinary least squares (OLS) regression by allowing the regression coefficients to vary continuously over the study area and generating a set of local-specific coefficients for each sample site [60–63]. Unlike the OLS model, which assumes stationarity across the study area and reflects the “average” or “global” condition of the relationship, the output of the GWR model has proven to be an effective visualization tool to understand spatially unstable variables [8,46,51,64]. Given its strong advantage, the GWR model has important implications for decision-making in ES management [19] and has been widely used to account for spatially nonstationary relationships between ESs and driving forces [8,51,64–67]. The GWR model can be expressed as follows [63]:

$$y_{i}=\beta_{0}(u_{i},v_{i})+\sum_{k=1}^{m}\beta_{k}(u_{i},v_{i})x_{ik}+\varepsilon_{i}$$ (8)

where y_i_ is the dependent variable of sample point i; (u_i_, v_i_) refers to the geographical coordinates (latitude and longitude) of sample point i; β_0_(u_i_, v_i_) is the intercept value for sample point i; β_k_ (u_i_, v_i_) denotes the local regression coefficient of independent variable x_ik_; and *ε*_i_ is the random error or residual for sample point i.

In the GWR model, the influence of each observation is determined by its geographic location and its distance from the observed points. Following earlier work, the Gaussian distance decay function was selected to express the spatial interactions as the decay function, which could be calculated by the following equation [68].

$$W_{\mathrm{ij}}=\exp\left(\frac{‐d^{2}{}_{\mathrm{ij}}}{h^{2}}\right)$$ (9)

where w_ij_ is the geographical weight of sample point j; d_ij_ is the distance between sample points i and j; and h is the kernel bandwidth, which controls the effect of the distance on the weight value. The golden selection search function was used to select the optimal bandwidth [69]. In MGWR 2.2.1 software, four driving forces (GDP, POP, runoff, and NDVI) were chosen as independent variables, and the biophysical values of two ESs (soil conservation and water yield) were chosen as dependent variables.

### 2.5 Scale effect analysis

**Fig 3:**
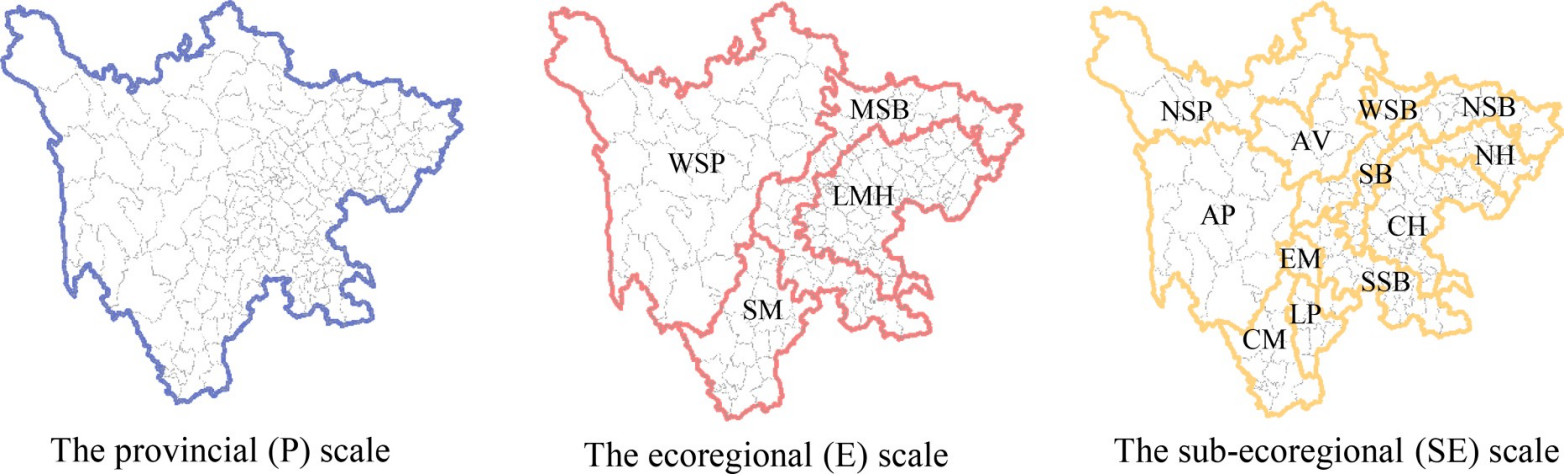
Vector range and unit divisions for the three different spatial scales analyzed in this study. (Reprinted from [Fig 3] under a CC BY license, with permission from [Resource and Environment Science and Data Center], original copyright [2018]).WSP: Western Sichuan Plateau; MSB: Mountains around Sichuan Basin; LMH: Low Mountains and Hills; SM: Southwestern Mountain; NSP: Northwest Sichuan Plateau; AP: Alpine Plateau; AV: Alpine Valley; NSB: Northern Sichuan Basin; WSB: Western Sichuan Basin; SSB: Southern Sichuan Basin; SB: Sichuan Basin; CH: Central Hill; NH: Northern Hill; EM: Emei Mountain; LP: Liangshan Plateau; CM: Central Mountain.

Based on the maneuverability of management policy, the differentiation laws in physical geography, and previous research, three spatial scales were chosen for this study [70]. The spatial distribution of the three spatial scales is shown in Fig 3.

Based on relevant research [3,67], the variations in the effects of drivers on ESs were calculated for each scale by the following steps. First, vector map layers were created at the ecoregion (E) and subecoregion (SE) scales using clip tools in ArcGIS 10.6 software (Esri, US). Thus, we were able to extract the statistical values of the regression coefficients of four driving forces (GDP, POP, runoff, and NDVI) from each of the four ecoregions and 12 subecoregions. Following this, the regression coefficients of each driving force within each vector map were then standardized to a fixed scale (for instance, 0–1 in this study) by the min-max normalization technique for convenient statistical analysis. Finally, the mean value and standard deviation of the regression coefficients of the four driving forces in each ecoregion and subecoregion were calculated using the Zonal Statistics tool in ArcGIS 10.6 software (Esri, US).

### 2.6 Analysis of environmental factors

**Finally**, canonical correlation analysis (CCA) was used to determine the potential correlations between environmental factors and the regression coefficients of driving forces at three spatial scales. As a multivariate statistical analysis, CCA is a standard tool used to determine the consistency (measured by correlation) between two sets of variables [71,72]. There is a rationale behind this method that is based on finding a pair of linear combinations of U_1-1_ and V_1-1_ (a linear combination of the dependent variables (X_1_, X_2_, …, X_k1_) and a linear combination of the independent variables (Y_1_, Y_2_, …, Y_k2_)) that have the largest correlation between U_1_ and V_1_. The process of finding a pair of linear combinations of U_1-2_…U_1-d_ and V_1-2_…V_1-d_ continues until no correlation between U_1_ and V_1_ remains statistically significant [10]. CCA represents a high-dimensional correlation between the variables (X_1_, X_2_, …, X_k1_) and (Y_1_, Y_2_, …, Y_k2_) with a few pairs of canonical variables. In this study, the absolute values of canonical loadings from small to large indicate the internal relationships between environmental factors, and the regression coefficients of driving forces range from weak to strong. CCA was performed using SPSS 26.0.

## 3 Results

### 3.1 Spatial distribution of ecosystem services

The distribution of soil conservation (SC) and water yield (WY) is uneven in Sichuan Province, and there are significant differences in the distribution between regions. Low values of SC mean that ecosystems are less capable of reducing soil erosion and mitigating sedimentation in rivers, lakes and reservoirs, while high values of SC mean that this capability is greater. The low values of SC are mostly located in the southeastern and northwestern parts of Sichuan, particularly in the Northwest Sichuan Plateau and Central Hill, while the high values are mainly concentrated in northeastern Sichuan, including the Northern Sichuan Basin and Northern Hill.

Low WY values imply a smaller amount of water flowing out of a drainage basin within a specified period of time, while high WY values imply a larger amount of water flowing out of a drainage basin within a specified period of time. WY values are low in northwestern Sichuan (e.g., Northwest Sichuan Plateau, Alpine Plateau), while high values are mainly found in northeastern Sichuan, particularly in the Mountains around the Sichuan Basin and Low mountains and hills. The distribution of WY in Sichuan Province displays a decreasing trend from southeast to northwest, as well as strong geomorphological distribution characteristics.

### 3.2 Spatial variations in the effects of driving forces on ecosystem services

**Fig 4:**
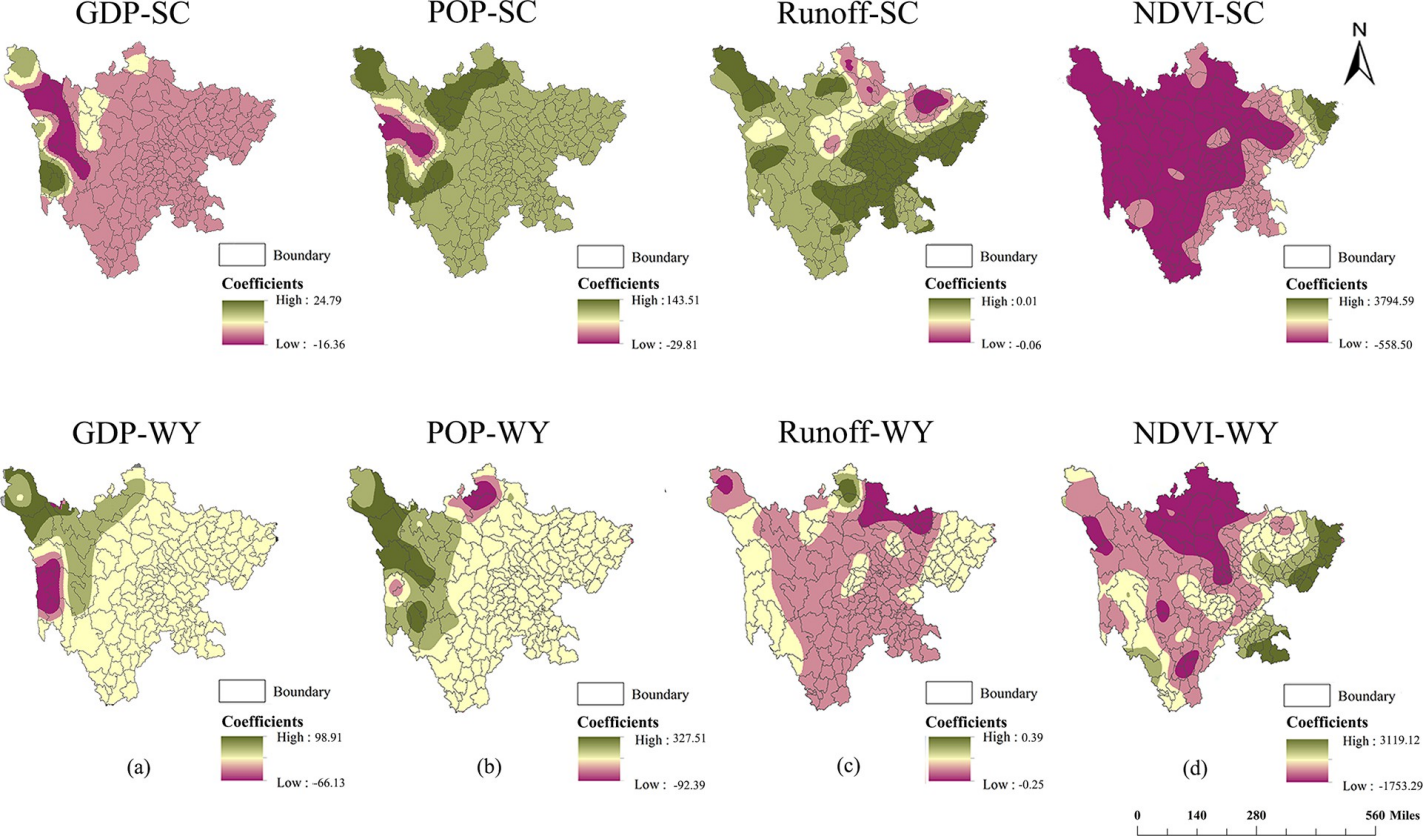
Spatial distribution of local regression coefficients between soil conservation (SC)/water yield (WY) and four drivers: Gross domestic production (GDP), population density (POP), Runoff, and NDVI.

The spatial variations in the effects of driving forces on ESs were identified by mapping the local regression coefficients of each driving force from the GWR model using ArcGIS 10.6 software (Esri, US). Overall, Fig 4 demonstrates that the four driving forces (GDP, POP, runoff, and NDVI) have significant positive and negative impacts on soil conservation and water yield, and their effects on the two services vary spatially. Fig 4A and 4B shows the effects of GDP and POP on ecosystem services. Both stronger positive and negative impacts of GDP and POP on soil conservation and water yield are mainly observed in the Western Sichuan Plateau. Fig 4(C) shows the effects of runoff on ecosystem services. A stronger positive impact of runoff on soil conservation is observed in the southeast of the province, mainly in Central Hill and Northern Hill, while negative effects of runoff on soil conservation and water yield are mainly found in the northeast fringe. Fig 4(D) shows the effects of NDVI on ecosystem services. NDVI has a significant negative effect on soil conservation in the central and western parts of the province and a positive effect on soil conservation and water yield on the east side of the province.

### 3.3 Scale dependency of driving forces

The variations in the statistical values of the regression coefficient of four drivers within each ecoregion or subregion are shown in Tables 2 and 3. Statistical results indicate that the impacts of driving forces (GDP, POP, runoff, and NDVI) on two services (soil conservation and water yield) differ significantly across three spatial scales (provincial, ecoregional, and subecoregional). In general, regional differences may be small within ecoregions or large between them. However, within some ecoregions (e.g., the Western Sichuan Plateau), there are also differences in the driving force coefficients. In the Southwestern Mountain, the driving coefficients of soil conservation vary significantly between subecoregions, while those of water yield are not significantly different.

**Table 2:** Statistical summary of the local regression coefficients of soil conservation at the provincial (P) scale, ecoregion (E) scale, and subecoregion (SE) scale (p < 0.05).

	Scale		Runoff		POP		GDP		NDVI	
P	E	SE	Mean	Std. Deviation	Mean	Std. Deviation	Mean	Std. Deviation	Mean	Std. Deviation
P			0.70	0.02	0.40	0.03	0.39	0.05	0.58	0.04
	WSP		0.60	0.02	0.39	0.03	0.29	0.05	0.61	0.04
		NSP	0.52	0.04	0.42	0.05	0.42	0.07	0.46	0.08
		AV	0.64	0.02	0.28	0.05	0.43	0.05	0.69	0.05
		AP	0.70	0.03	0.41	0.06	0.38	0.08	0.39	0.10
	SM		0.37	0.02	0.53	0.02	0.62	0.03	0.40	0.04
		CM	0.66	0.05	0.70	0.05	0.62	0.04	0.39	0.06
		LP	0.20	0.02	0.59	0.06	0.56	0.04	0.54	0.05
		EM	0.58	0.01	0.35	0.02	0.55	0.03	0.37	0.02
	LMH		0.44	0.02	0.37	0.04	0.34	0.01	0.58	0.03
		SSB	0.39	0.04	0.45	0.06	0.31	0.04	0.59	0.05
		WSB	0.45	0.03	0.43	0.03	0.34	0.03	0.42	0.05
		NSB	0.47	0.04	0.59	0.06	0.44	0.06	0.33	0.06
	MSB		0.51	0.01	0.43	0.02	0.6	0.02	0.45	0.04
		SB	0.51	0.02	0.37	0.06	0.60	0.05	0.45	0.06
		CH	0.53	0.04	0.48	0.05	0.32	0.05	0.48	0.07
		NH	0.61	0.02	0.38	0.04	0.57	0.04	0.44	0.03

WSP: Western Sichuan Plateau; MSB: Mountains around Sichuan Basin; LMH: Low mountains and Hills; SM: Southwestern Mountain; NSP: Northwest Sichuan Plateau; AP: Alpine plateau; AV: Alpine valley; NSB: Northern Sichuan Basin; WSB: Western Sichuan Basin; SSB: Southern Sichuan Basin; SB: Sichuan Basin; CH: Central Hill; NH: Northern Hill; EM: Emei Mountain; LP: Liangshan Plateau; CM: Central Mountain.

**Table 3:** Statistical summary of the local regression coefficients of water yield at the provincial (P) scale, ecoregion (E) scale, and subecoregion (SE) scale (p < 0.05).

	Scale		Runoff		POP		GDP		NDVI	
P	E	SE	Mean	Std. Deviation	Mean	Std. Deviation	Mean	Std. Deviation	Mean	Std. Deviation
P			0.68	0.03	0.69	0.02	0.44	0.02	0.52	0.05
	WSP		0.67	0.03	0.5	0.05	0.44	0.07	0.51	0.09
		NSP	0.60	0.05	0.26	0.09	0.36	0.09	0.54	0.10
		AV	0.42	0.03	0.39	0.09	0.22	0.05	0.57	0.12
		AP	0.75	0.03	0.67	0.05	0.44	0.07	0.49	0.08
	SM		0.82	0.05	0.40	0.08	0.37	0.06	0.61	0.10
		CM	0.84	0.05	0.42	0.07	0.32	0.05	0.63	0.09
		LP	0.76	0.04	0.47	0.05	0.42	0.05	0.50	0.08
		EM	0.85	0.03	0.39	0.03	0.40	0.04	0.51	0.06
	LMH		0.48	0.05	0.39	0.04	0.3	0.04	0.55	0.08
		SSB	0.51	0.03	0.51	0.04	0.80	0.04	0.52	0.06
		WSB	0.77	0.61	0.44	0.04	0.39	0.04	0.46	0.06
		NSB	0.30	0.03	0.61	0.05	0.36	0.05	0.41	0.05
	MSB		0.67	0.04	0.51	0.07	0.44	0.07	0.38	0.11
		SB	0.70	0.02	0.47	0.05	0.46	0.05	0.43	0.08
		CH	0.67	0.03	0.51	0.43	0.40	0.05	0.38	0.09
		NH	0.54	0.43	0.65	0.04	0.45	0.04	0.44	0.08

WSP: Western Sichuan Plateau; MSB: Mountains around Sichuan Basin; LMH: Low mountains and Hills; SM: Southwestern Mountain; NSP: Northwest Sichuan Plateau; AP: Alpine plateau; AV: Alpine valley; NSB: Northern Sichuan Basin; WSB: Western Sichuan Basin; SSB: Southern Sichuan Basin; SB: Sichuan Basin; CH: Central Hill; NH: Northern Hill; EM: Emei Mountain; LP: Liangshan Plateau; CM: Central Mountain.

### 3.4 Relationships between environmental factors and drivers

Based on the absolute values of canonical loadings from CCA, the associations between environmental factors and driver regression coefficients were identified across three spatial scales (Tables 4 and 5). According to previous research [10], we chose a significance level of 0.05 and excluded correlations that were not significant at this level.

**Table 4:** The associations between environmental factors and the local regression coefficients of soil conservation at the provincial (P) scale, ecoregion (E) scale, and subecoregion (SE) scale (p < 0.05).

Scale			Physical factor				Socioeconomic factors		Topographical variables			Biophysical factors
			PBL	PCL	PGL	PFL	GDP	POP	DEM	Slope	RDLS	NDVI
P					✔				✔		✔	
	WSP					✔		✔	✔		✔T	✔
		NSP				✔	✔					✔
		AV			✔						✔	
		AP			✔	✔		✔	✔		✔	
	SM					✔			✔		✔	
		CM			✔				✔	✔		✔
		LP				✔			✔		✔	✔
		EM					✔	✔			✔	
	MSB			✔					✔			
		SSB		✔			✔				✔	
		WSB		✔	✔			✔	✔			✔
		NSB				✔	✔		✔			
	LMH			✔					✔		✔	
		SB			✔		✔	✔				✔
		CH		✔		✔	✔					
		NH	✔	✔					✔		✔	

PBL: The proportion of built-up land; PCL: The proportion of cropland; PGL: The proportion of grassland; PFL: The proportion of forestland; GDP: Gross domestic product; POP: Population density; DEM: Digital Elevation Model; RDLS: The relief degree of the land surface; NDVI: Normalized Difference Vegetation Index. WSP: Western Sichuan Plateau; MSB: Mountains around Sichuan Basin; LMH: Low mountains and Hills; SM: Southwestern Mountain; NSP: Northwest Sichuan Plateau; AP: Alpine plateau; AV: Alpine valley; NSB: Northern Sichuan Basin; WSB: Western Sichuan Basin; SSB: Southern Sichuan Basin; SB: Sichuan Basin; CH: Central Hill; NH: Northern Hill; EM: Emei Mountain; LP: Liangshan Plateau; CM: Central Mountain.

**Table 5:** The associations between environmental factors and the local regression coefficients of water yield at the provincial (P) scale, ecoregion (E) scale, and subecoregion (SE) scale (p < 0.05).

Scale			Physical factor				Socioeconomic factors		Topographical variables			Biophysical factors
			PBL	PCL	PGL	PFL	GDP	POP	DEM	Slope	RDLS	NDVI
P			✔						✔		✔	✔
	WSP					✔		✔	✔			✔
		NSP				✔	✔				✔	
		AV		✔	✔	✔			✔		✔	
		AP			✔	✔					✔	✔
	SM					✔			✔		✔	✔
		CM			✔				✔		✔	
		LP				✔			✔		✔	
		EM					✔		✔		✔	✔
	MSB			✔			✔				✔	
		SSB		✔			✔		✔		✔	
		WSB		✔					✔			
		NSB	✔			✔						
	LMH		✔	✔			✔		✔		✔	
		SB			✔		✔					✔
		CH		✔		✔	✔					✔
		NH							✔		✔	

PBL: The proportion of built-up land; PCL: The proportion of cropland; PGL: The proportion of grassland; PFL: The proportion of forestland; GDP: Gross domestic product; POP: Population density; DEM: Digital Elevation Model; RDLS: The relief degree of the land surface; NDVI: Normalized Difference Vegetation Index. WSP: Western Sichuan Plateau; MSB: Mountains around Sichuan Basin; LMH: Low mountains and Hills; SM: Southwestern Mountain; NSP: Northwest Sichuan Plateau; AP: Alpine plateau; AV: Alpine valley; NSB: Northern Sichuan Basin; WSB: Western Sichuan Basin; SSB: Southern Sichuan Basin; SB: Sichuan Basin; CH: Central Hill; NH: Northern Hill; EM: Emei Mountain; LP: Liangshan Plateau; CM: Central Mountain.

At the provincial (P) scale, the key factors underlying the regression coefficients of drivers are the relief degree of the land surface, digital elevation model, proportion of grassland, normalized difference vegetation index, and proportion of built-up land.

At the ecoregional (E) scale, the major factors correlating the regression coefficients of drivers are population density, the proportion of forestland, digital elevation model, the relief degree of the land surface, and normalized difference vegetation index in the Western Sichuan Plateau (WSP); the relief degree of the land surface, the proportion of forestland, normalized difference vegetation index, and digital elevation model in the Southwestern Mountain (SM); the proportion of cropland, digital elevation model, the relief degree of the land surface, and gross domestic product in the Mountains around the Sichuan Basin (MSB); and the proportion of cropland, digital elevation model, the relief degree of the land surface, gross domestic product, and the proportion of built-up land in the Low mountains and Hills (LMH).

At the subecoregional (SE) scale, the primary factors underlying the regression coefficients of drivers are the normalized difference vegetation index, the proportion of forestland, proportion of built-up land, the relief degree of the land surface, and gross domestic product in the Northwest Sichuan Plateau (NSP); the proportion of grassland, the relief degree of the land surface, digital elevation model, and the proportion of forestland in the Alpine valley (AV); digital elevation model, the proportion of forestland, population density, normalized difference vegetation index, the proportion of grassland, and the relief degree of the land surface in the Alpine plateau (AP); digital elevation model, normalized difference vegetation index, slope, the relief degree of the land surface, and the proportion of grassland in the Central Mountain (CM); the relief degree of the land surface, the proportion of forestland, normalized difference vegetation index, and digital elevation model in the Liangshan Plateau; gross domestic product, population density, normalized difference vegetation index, the relief degree of the land surface, and digital elevation model in the Emei Mountain (EM); digital elevation model, the proportion of cropland, the relief degree of the land surface, and gross domestic product in the Southern Sichuan Basin (SSB); normalized difference vegetation index, the proportion of cropland, population density, the proportion of grassland, and digital elevation model in the Western Sichuan Basin (WSB); the proportion of built-up land, gross domestic product, the proportion of forestland, and digital elevation model in the Northern Sichuan Basin (NSB); population density, normalized difference vegetation index, the proportion of grassland, and gross domestic product in the Sichuan Basin (SB); the proportion of forestland, normalized difference vegetation index, gross domestic product, and the proportion of cropland in the Central Hill (CH); and the proportion of built-up land, the relief degree of the land surface, the proportion of cropland, and digital elevation model in the Northern Hill (NH).

Briefly, as shown in Tables 4–6, it is evident that the major factors associated with the regression coefficients of drivers are significantly different at the three spatial scales (provincial, ecoregional, and subecoregional) in Sichuan Province. Specifically, physical, topographical, and biophysical factors were key factors associated with the variations in the relationship between ESs and drivers at the provincial scale, and physical, socioeconomic, topographical, and biophysical factors all contributed to these changes at the ecoregional and subecoregional scales.

**Table 6:** The relationship between soil conservation (SC) and water yield (WY) at three spatial levels. “+” represents synergy, and “−” signifies a trade-off.

Scales	Relationship between SC and WY			
P scale	+			
E scale	WSP +	RMS +	BMA +	BHA +
SE scale	NP + AV + AP −	KM + LP + EM +	SB + WB − NB +	PM + PH + PP +

WSP: Western Sichuan Plateau; MSB: Mountains around Sichuan Basin; LMH: Low mountains and Hills; SM: Southwestern Mountain; NSP: Northwest Sichuan Plateau; AP: Alpine plateau; AV: Alpine valley; NSB: Northern Sichuan Basin; WSB: Western Sichuan Basin; SSB: Southern Sichuan Basin; SB: Sichuan Basin; CH: Central Hill; NH: Northern Hill; EM: Emei Mountain; LP: Liangshan Plateau; CM: Central Mountain.

## 4 Discussion

### 4.1 Scale dependency of driving forces

There has been some evidence that the impacts of drivers on ESs may vary with scale [6,7,20]. Based on Tables 2 and 3, the scale dependency of the impacts of driving forces on the two services was confirmed in this study. The reason for this shift is that landscape multifunctionality is more evident in larger spatial units than in smaller ones, facilitating a shift in the relationship between services from trade-offs to synergies [3,31,42]. Xu et al. 2017 [5] found that the ES of food supply and tourism resulted in trade-offs at fine spatial scales (<7 km); however, such trade-offs became synergies at a coarser spatial scale (>7 km). Our research also found the same phenomenon between soil conservation and water yield, where this relationship changed from trade-off (at the SE scale) to synergy (at the P and E scales) (Table 6). The study supports the hypothesis that an increase in the spatial scale of observation will result in a homogenization of the landscape [42].

### 4.2 Relationships between environmental factors and drivers

#### Sichuan Province

The results in Table 4 reveal that at the provincial (P) scale, the digital elevation model and the relief degree of the land surface were the key factors associated with the variations in the regression coefficients of the four driving forces in Sichuan Province. Topography, as a fundamental physical element, plays an essential role in determining the distribution of land use [8,73,74], human activities [26,75], vegetation [76–78], and water resources [79] across the world. In Sichuan Province, there are a plethora of landform types, including plateaus, mountains, hills, basins, and plains. In the east, there are basins and hills, with elevations mostly between 1000 and 3000 m. In the west, there are high mountain plateaus and mountainous landscapes, with elevations mostly above 4000 m. Huge differences in topographical conditions first cause variations in environmental aspects (e.g., temperature, precipitation, wind speed) and then result in remarkable regional social-ecological differences [44]. As shown in Fig 2, the four drivers in Sichuan Province are extremely unevenly distributed. For example, the highest mean values of population density and gross domestic product are mostly located in the Sichuan Basin, and the lowest values are mainly concentrated in the Northwest Sichuan Plateau (Table 7). The spatial distribution of the main LULC types exhibited regional differences in Sichuan Province [33]. Grassland and woodland are mainly distributed in western areas, while cropland and construction land areas are mainly concentrated in the eastern and southeastern regions. In addition, the spatial distribution of vegetation is also closely associated with topography: farmland and human settlements are usually found on flat terrain, while forested areas occupy steeper slopes in Sichuan Province. Hence, these results suggest that significant differences in topographic conditions can be taken into account when exploring the influences of drivers on ESs and optimizing ecological management in Sichuan Province.

**Table 7:** Statistical summary of the mean values of the four drivers at the provincial (P) scale, ecoregion (E) scale, and subecoregion (SE) scale.

	Scale		GDP	POP	Runoff	NDVI
P	E	SE	Mean	Mean	Mean	Mean
P			626.92	169.94	372.64	0.77
	WSP		22.15	9.16	262.40	0.74
		NSP	8.14	6.85	116.79	0.78
		AV	18.93	8.61	400.29	0.76
		AP	50.84	14.25	168.25	0.70
	SM		394.41	105.71	646.04	0.82
		CM	572.10	114.28	992.81	0.81
		LP	157.78	99.27	38.37	0.81
		ME	190.41	89.80	470.33	0.84
	LMH		2476.52	634.04	436.61	0.76
		SSB	833.21	245.98	973.93	0.83
		WSB	747.53	195.92	104.91	0.82
		NSB	462.86	189.69	81.40	0.85
	MSB		691.94	212.54	428.05	0.84
		SB	7254.94	1169.23	979.33	0.70
		CH	1862.14	570.81	379.82	0.77
		NH	932.55	446.80	251.25	0.81

WSP: Western Sichuan Plateau; MSB: Mountains around Sichuan Basin; LMH: Low mountains and Hills; SM: Southwestern Mountain; NSP: Northwest Sichuan Plateau; AP: Alpine plateau; AV: Alpine valley; NSB: Northern Sichuan Basin; WSB: Western Sichuan Basin; SSB: Southern Sichuan Basin; SB: Sichuan Basin; CH: Central Hill; NH: Northern Hill; EM: Emei Mountain; LP: Liangshan Plateau; CM: Central Mountain.

#### Western Sichuan Plateau

Based on the results of Tables 5 and 6, the proportion of forestland, the proportion of grassland, and the normalized difference vegetation index are the main factors responsible for the variation in the regression coefficients of the four driving forces in the Western Sichuan Plateau. The reason may lie in the fact that grassland and woodland mostly cover western Sichuan Province (especially the Tibetan Autonomous Prefecture of Garz, Tibetan and Qiang Autonomous Prefecture of Ngawa, and Yi Autonomous Prefecture of Liangshan) and have important economic and ecological values [33]. On the one hand, woodland and grassland can provide humans with timber, energy, food, and other products and are the basis for local economic development and population agglomeration. On the other hand, natural ecosystems play a key role in local ecological conservation. For instance, surface runoff can be reduced due to the retention effect of the forest canopy and litter [38]. Therefore, significant differences in the economic and ecological values of local natural vegetation can be considered in managing the relationship between driving forces and ESs and in improving the localized landscape and ecosystem management in the Western Sichuan Plateau.

#### Southwestern Mountain

Based on Table 4, it is evident that the proportion of forestland, digital elevation model, and the relief degree of the land surface account for the majority of the variation in the regression coefficients of the four forces in the Southwestern Mountain. Southwest Sichuan is mainly covered by woodland and characterized by continuous karst topography, sheet erosion, and gully erosion [50,80]. Furthermore, West Sichuan is a region prone to natural hazards [44]. For example, the Central Mountain and Liangshan Plateau are areas with complex geological conditions, highly erodible water, and frequent natural disasters. Heavy precipitation and thin soil layers will increase the frequency of soil erosion, landslides, debris flows, and other natural hazards in the area. Such complex geological conditions will not only affect local forest growth and surface runoff but also hinder local economic development and human activities. Hence, significant differences in local complex geological conditions and natural disasters influence the change in driver impacts in the Southwestern Mountain.

#### Mountains around the Sichuan Basin

As shown in Table 4, the proportion of cropland and the relief degree of the land surface are the main factors responsible for the variation in the regression coefficients among the four driving forces in the Mountains around Sichuan Basin. Cropland is one of the dominant land classes in these regions. It is worth noting that the local farmland is mainly located on steep slopes, especially within the slope range of 15 to 35, as a result of the local topographical environment [80]. This phenomenon is more prevalent in regions such as Central Hill and Northern Hill. Slope farmland can lead to soil nutrient loss, soil structure deterioration, soil erosion, and increased surface runoff, which in turn pose a threat to local vegetation growth and economic and social development. Hence, the potential threat of sloping farmland to local drivers should be taken into account when managing the variations in driver influences in the Mountains around Sichuan Basin.

#### Low Mountains and Hills

Tables 5 and 6 indicate that the proportion of built-up land, the proportion of cropland, and the relief degree of the land surface contribute to the variations in the effects of drivers on ESs in the Low Mountains and Hills. Since 1991, Sichuan Province has experienced rapid economic development and urban expansion. This phenomenon was prominent in the Low Mountains and Hills, where the velocity of economic development is faster than in other places due to the relatively flat terrain. As a result of economic growth and urbanization, the area has expanded significantly in terms of both built-up land and cropland [33]. During this process, extensive highly artificially modified land may alter the hydrological flow path and soil properties and have long-term consequences on local surface runoff and plant growth [81]. Therefore, significant differences in urbanization levels should be considered when exploring and managing the relationship between ESs and driving forces and promoting ecological sustainability in the Low Mountains and Hills.

### 4.3 Implication

The goal of environmental management is the sustainable utilization of ecosystems to meet human demand [82]. Numerous studies have explored regional management based on the biophysical or economic values of ESs; however, these studies have paid less attention to the regional differences in the effects of driving forces on ESs. According to the results of CCA, the relationship between drivers and ESs varied at different spatial scales, with corresponding shifts in the associated environmental factors within each ecoregion. Therefore, in highly heterogeneous areas, the overall policy is difficult to implement [19]. It is important to fully understand the associations between drivers and ESs across multiple spatial scales and take into account scale-related information to optimize localized ecological management [6]. According to our results, a highly heterogeneous region should be able to incorporate significant differences in topographical conditions (e.g., altitude, slope) when exploring the relationship between drivers and ESs and determining the appropriate environmental management strategy at the provincial level, while differences in physical and socioeconomic conditions (e.g., urbanization level, human activity, local vegetation coverage) should be considered at the ecoregional and subecoregional levels.

More specifically, our results suggest that in Sichuan Province, topographical factors (e.g., the relief degree of the land surface, digital elevation model) should be considered as ecoregion boundaries to facilitate localized natural conservation, forestry management and ecological management. In the Western Sichuan Plateau, the focus of ecological management should be on protecting and recovering local natural vegetation and coordinating the relationship between socioeconomic development and ecological management. In response, a series of ecological restoration projects, such as the “Grain for Green Program (GFGP)” and “Natural Forest Conservation Program (NFCP)”, could be implemented in an effort to control and mitigate the degradation of the local environment. In the Southwestern Mountain, managers should prioritize optimizing the coverage and structure of local forests to improve their ecological functions and maintain the sustainability of the local environment. With respect to local complex geological conditions, ecological programs for rocky desertification and soil erosion should be combined to enhance the quality, productivity, and diversity of local ecosystems. In the Mountains around Sichuan Basin, managers should be concerned about the potential threat of local sloping farmland. The implementation of soil erosion control measures (e.g., protection of the topsoil layer, application of organic fertilizer and renovation of sloping farmland) and forest conservation projects (e.g., returning farmland to forest, natural forest protection measures, prohibition of timber harvesting) should be coordinated to enhance the resistance of local ecosystems to water erosion [82]. In the Low Mountains and Hills, local managers should be aware of the negative effects of urbanization and the importance of reasonable urban planning. Improving forest coverage and protecting ecological functional zones are encouraged to optimize the local ecological environment and promote regional sustainable development [83]. In addition, we suggest the need to develop agroforestry to ensure supply services while guaranteeing the integrity and diversity of the region’s ecosystems.

## Conclusions

Based on analyzing the changes in the effects of driving forces on ESs at three spatial scales, identifying the main factors associated with these variations at each spatial scale, and discussing the implications of localized ecological management by analyzing the relationships between environmental factors and drivers, this study examines the significance of the scale dependency of drivers on ESs for localized ecological management. According to these findings, GDP, POP, runoff, and NDVI all have different effects on soil conservation and water yield at three spatial scales (provincial, ecoregional, and subecoregional scales) in Sichuan Province. The results of the CCA indicated that the major factors associated with the regression coefficients of drivers are significantly different at the three spatial scales (provincial, ecoregional, and subecoregional scales). At the provincial (P) scale, physical, topographical, and biophysical factors were the principal factors associated with the variation in the local regression coefficients of the four driving forces. At the ecoregional and subecoregional scales, physical, socioeconomic, topographical, and biophysical factors all contribute to these variations. Our results suggest that significant differences in topographical conditions (e.g., altitude, slope) can be incorporated for exploring the relationship between drivers and ESs and optimizing ecological management at the provincial scale, whereas significant differences in physical and socioeconomic conditions (e.g., urbanization levels, human activity, local vegetation coverage) are more meaningful for localized management at the ecoregional and subecological scales. These findings highlight the importance of awareness among scientists and decision-makers of scale issues of the spatially varying relationships between driving forces and ESs and provide scale-based references for local policy-makers to optimize ecological management at different scales.

## References

[1] Assessment M. Ecosystems and human well-being: synthesis. MEA. 2005.

[2] Costanza R, Arge, Groot RD, Farberk S, Belt M. The value of the world’s ecosystem services and natural capital. Nature. 1997;387(15):253–60. 10.1016/S0921-8009(98)00020-2.

[3] Dou H, Li X, Li S, Dang D. How to Detect Scale Effect of Ecosystem Services Supply? A Comprehensive Insight from Xilinhot in Inner Mongolia, China. Sustainability. 2018;10. (10). 10.3390/su10103654.

[4] Bai Y, Chen Y, Alatalo JM, Yang Z, Jiang B. Scale effects on the relationships between land characteristics and ecosystem services- a case study in Taihu Lake Basin, China. Sci Total Environ. 2020;716:137083. doi: 10.1016/j.scitotenv.2020.137083 32036149

[5] Xu S, Liu Y, Wang X, Zhang G. Scale effect on spatial patterns of ecosystem services and associations among them in semi-arid area: A case study in Ningxia Hui Autonomous Region, China. Science of The Total Environment. 2017;598:297–306. doi: 10.1016/j.scitotenv.2017.04.009 28445827

[6] Shen J, Li S, Liu L, Liang Z, Wang Y, Wang H, et al. Uncovering the relationships between ecosystem services and social-ecological drivers at different spatial scales in the Beijing-Tianjin-Hebei region. Journal of Cleaner Production. 2021;290:125193. 10.1016/j.jclepro.2020.125193.

[7] Cui F, Tang H, Zhang Q, Wang B, Dai L. Integrating ecosystem services supply and demand into optimized management at different scales: A case study in Hulunbuir, China. Ecosystem Services. 2019;39:100984. 10.1016/j.ecoser.2019.100984.

[8] Ajaz Ahmed MA, Abd-Elrahman A, Escobedo FJ, Cropper WP, Martin TA, Timilsina N. Spatially-explicit modeling of multi-scale drivers of aboveground forest biomass and water yield in watersheds of the Southeastern United States. Journal of Environmental Management. 2017;199:158–71. doi: 10.1016/j.jenvman.2017.05.013 28531796

[9] Burkhard B, Kroll F, Nedkov S, Müller F. Mapping ecosystem service supply, demand and budgets. Ecological Indicators. 2012;21:17–29. 10.1016/j.ecolind.2011.06.019.

[10] Su C, Dong M, Fu B-J, Liu G-h. Scale effects of sediment retention, water yield and NPP: a case study of the Chinese Loess Plateau. Land Degradation & Development. 2019;31. 10.1002/ldr.3536.

[11] Syrbe R-U, Walz U. Spatial indicators for the assessment of ecosystem services: Providing, benefiting and connecting areas and landscape metrics. Ecological Indicators. 2012;21:80–8. 10.1016/j.ecolind.2012.02.013.

[12] Zhang Y, Liu Y, Pan J, Zhang Y, Liu D, Chen H, et al. Exploring Spatially Non-Stationary and Scale-Dependent Responses of Ecosystem Services to Urbanization in Wuhan, China. Int J Environ Res Public Health. 2020;17. (9). doi: 10.3390/ijerph17092989 32344851 PMC7246692

[13] Gomes E, Inácio M, Bogdzevič K, Kalinauskas M, Karnauskaitė D, Pereira P. Future land-use changes and its impacts on terrestrial ecosystem services: A review. Science of The Total Environment. 2021;781:146716. doi: 10.1016/j.scitotenv.2021.146716 33798896

[14] Pereira P. Ecosystem services in a changing environment. Science of The Total Environment. 2020;702:135008. doi: 10.1016/j.scitotenv.2019.135008 31733548

[15] Chen W, Chi G, Li J. Ecosystem Services and Their Driving Forces in the Middle Reaches of the Yangtze River Urban Agglomerations, China. Int J Environ Res Public Health. 2020;17. (10). doi: 10.3390/ijerph17103717 32466142 PMC7277137

[16] Han Z, Song W, Deng X, Xu X. Trade-Offs and Synergies in Ecosystem Service within the Three-Rivers Headwater Region, China. Water. 2017;9(8):588. 10.3390/w9080588.

[17] Khaledian Y, Kiani F, Ebrahimi S, Brevik E, Aitkenhead-Peterson J. Assessment and Monitoring of Soil Degradation during Land Use Change Using Multivariate Analysis. Land Degradation and Development. 2017;28:128–41. 10.1002/ldr.2541.

[18] Sannigrahi S, Qi Z, Francesco P, Kumar JP, Bidroha B, Saskia K, et al. Responses of ecosystem services to natural and anthropogenic forcings: A spatial regression based assessment in the world’s largest mangrove ecosystem. The Science of the Total Environment. 2020;715(May1):137004.1–.13. 10.1016/j.scitotenv.2020.137004. 32045970

[19] Sun X, Tang H, Yang P, Hu G, Liu Z, Wu J. Spatiotemporal patterns and drivers of ecosystem service supply and demand across the conterminous United States: A multiscale analysis. Sci Total Environ. 2020;703:135005. doi: 10.1016/j.scitotenv.2019.135005 31733497

[20] Tolessa T, Senbeta F, Kidane M. The impact of land use/land cover change on ecosystem services in the central highlands of Ethiopia. Ecosystem Services. 2017;23:47–54. 10.1016/j.ecoser.2016.11.010.

[21] Wang H, Liu G, Li Z, Zhang L, Wang Z. Processes and driving forces for changing vegetation ecosystem services: Insights from the Shaanxi Province of China. Ecological Indicators. 2020;112. 10.1016/j.ecolind.2020.106105.

[22] Zhang Li, Buyantuev Bao, Zhang. How Do Trade-Offs and Synergies between Ecosystem Services Change in the Long Period? The Case Study of Uxin, Inner Mongolia, China. Sustainability. 2019;11. (21). 10.3390/su11216041.

[23] Song W, Deng X, Yuan Y, Wang Z, Li Z. Impacts of land-use change on valued ecosystem service in rapidly urbanized North China Plain. Ecological Modelling. 2015;318:245–53. 10.1016/j.ecolmodel.2015.01.029.

[24] Asmus ML, Nicolodi J, Anello LS, Gianuca K. The risk to lose ecosystem services due to climate change: A South American case. Ecological Engineering. 2019;130:233–41. 10.1016/j.ecoleng.2017.12.030.

[25] Hasan SS, Lin Z, Miah MG, Ahamed T, Samie A. Impact of land use change on ecosystem services: A review. Environmental Development. 2020;34:100527. 10.1016/j.envdev.2020.100527.

[26] Chen W, Chi G, Li J. The spatial aspect of ecosystem services balance and its determinants. Land Use Policy. 2020;90. 10.1016/j.landusepol.2019.104263.

[27] Liu Y, Li J, Zhang H. An ecosystem service valuation of land use change in Taiyuan City, China. Ecological Modelling. 2012;225:127–32. 10.1016/j.ecolmodel.2011.11.017.

[28] Hein L, van Koppen K, de Groot RS, van Ierland EC. Spatial scales, stakeholders and the valuation of ecosystem services. Ecological Economics. 2006;57(2):209–28. 10.1016/j.ecolecon.2005.04.005.

[29] Martín-López B, Gómez-Baggethun E, Lomas PL, Montes C. Effects of spatial and temporal scales on cultural services valuation. Journal of Environmental Management. 2009;90(2):1050–9. doi: 10.1016/j.jenvman.2008.03.013 18486302

[30] Graham LJ, Eigenbrod F. Scale dependency in drivers of outdoor recreation in England. People and Nature. 2019;1(3):406–16. 10.1002/pan3.10042.

[31] Raudsepp-Hearne C, Peterson GDJE, Society. Scale and ecosystem services: how do observation, management, and analysis shift with scale—lessons from Québec. Ecology & Society. 2016;21(3). 10.5751/ES-08605-210316.

[32] Hou Y, Zhao W, Liu Y, Yang S, Hu X, Cherubini F. Relationships of multiple landscape services and their influencing factors on the Qinghai–Tibet Plateau. Landscape Ecology. 2021;36(7):1987–2005. 10.1007/s10980-020-01140-3.

[33] Fan M, Chen L. Spatial characteristics of land uses and ecological compensations based on payment for ecosystem services model from 2000 to 2015 in Sichuan Province, China. Ecological Informatics. 2019;50:162–83. 10.1016/j.ecoinf.2019.01.001.

[34] de Groot RS, Alkemade R, Braat L, Hein L, Willemen L. Challenges in integrating the concept of ecosystem services and values in landscape planning, management and decision making. Ecological Complexity. 2010;7(3):260–72. 10.1016/j.ecocom.2009.10.006.

[35] Wu J, Jones K, Li H-t, Loucks O. Scaling and Uncertainty Analysis in Ecology: Methods and Applications 2006.

[36] Yao J, He X, Chen W, Ye Y, Guo R, Yu LJES. A local-scale spatial analysis of ecosystem services and ecosystem service bundles in the upper Hun River catchment, China. 2016;22:104–10.

[37] Felipe-Lucia, María, R., Comín, Francisco A., et al. Interactions Among Ecosystem Services Across Land Uses in a Floodplain Agroecosystem. 2014. doi: 10.5751/ES-06249-190120

[38] Hao R, Yu D, Wu J. Relationship between paired ecosystem services in the grassland and agro-pastoral transitional zone of China using the constraint line method. Agriculture, Ecosystems & Environment. 2017;240:171–81. 10.1016/j.agee.2017.02.015.

[39] Hou Y, Lü Y, Chen W, Fu B. Temporal variation and spatial scale dependency of ecosystem service interactions: a case study on the central Loess Plateau of China. Landscape Ecology. 2017;32(6):1201–17. 10.1007/s10980-017-0497-8.

[40] Hu T, Wu J, Li W. Assessing relationships of ecosystem services on multi-scale: A case study of soil erosion control and water yield in the Pearl River Delta. Ecological Indicators. 2019;99:193–202. 10.1016/j.ecolind.2018.11.066.

[41] Yue TX, Liu JY, Li ZQ, Chen SQ, Ma SN, Tian YZ, et al. Considerable effects of diversity indices and spatial scales on conclusions relating to ecological diversity. Ecological Modelling. 2005;188(2):418–31. 10.1016/j.ecolmodel.2004.12.019.

[42] Madrigal-Martínez S, Miralles i García J. Assessment Method and Scale of Observation Influence Ecosystem Service Bundles. Land. 2020;9:392. 10.3390/land9100392.

[43] Mehring M, Ott E, Hummel D. Ecosystem services supply and demand assessment: Why social-ecological dynamics matter. Ecosystem Services. 2018;30:124–5. 10.1016/j.ecoser.2018.02.009.

[44] Liu Y, Yang S, Han C, Ni W, Zhu Y. Variability in Regional Ecological Vulnerability: A Case Study of Sichuan Province, China. International Journal of Disaster Risk Science. 2020;11(6):696–708. 10.1007/s13753-020-00295-6.

[45] Rao E, Xiao Y. Spatial characteristics and effects of soil conservation service in Sichuan Province. Acta Ecologica Sinica. 2018;38(24):8741–9 (in Chinese). 10.5846/stxb201806011217.

[46] He X, Mai X, Shen G. Poverty and Physical Geographic Factors: An Empirical Analysis of Sichuan Province Using the GWR Model. 2021;13(1):100.

[47] Food, Agriculture Organization of the United Nations (F, International Institute for Applied Systems A. China soil map based harmonized world soil database (HWSD) (v1.1) (2009). In: National Tibetan Plateau Data C, editor.: National Tibetan Plateau Data Center; 2019.

[48] About ArcGIS | Mapping & Analytics Platform. Available online: https://www.esri.com/en-us/arcgis/ about-arcgis/overview.

[49] Cheng M, Huang B, Kong L, Ouyang Z. Ecosystem Spatial Changes and Driving Forces in the Bohai Coastal Zone. Int J Environ Res Public Health. 2019;16, 536. (4). doi: 10.3390/ijerph16040536 30781813 PMC6406312

[50] Li R, Bennett J, Wang X. Predicting environmental impacts for assessing land use change options in Sichuan Province, China. Land Use Policy. 2013;30(1):784–90. 10.1016/j.landusepol.2012.05.019.

[51] Yang M, Gao X, Zhao X, Wu P. Scale effect and spatially explicit drivers of interactions between ecosystem services—A case study from the Loess Plateau. Science of The Total Environment. 2021;785:147389. 10.1016/j.scitotenv.2021.147389.

[52] Zhu Z, Hou L. Shortage appraisal and sustainable utilization of water resource in Sichuan province. SCIENCE OF SOIL AND WATER CONSERVATION. 2006;4(4):92–5 (in Chinese). 10.3969/j.issn.1672-3007.2006.04.017.

[53] Liu C, Yang M, Hou Y, Xue X. Ecosystem service multifunctionality assessment and coupling coordination analysis with land use and land cover change in China’s coastal zones. Science of The Total Environment. 2021;797:149033. doi: 10.1016/j.scitotenv.2021.149033 34303237

[54] Renard KG, Foster GR, Weesies GA, Porter JP. RUSLE: Revised universal soil loss equation. J Soil & Water Conservation. 1991;46. (1). 10.1002/9781444328455.ch8.

[55] Wischmerie WH, Smith DD. Predicting rainfall-erosion losses from cropland east of the Rocky Mountains: a guide to conservation planning. Agric Hardbook. 1965;282:1–17.

[56] Sharpley AN, Williams JR. EPIC-erosion/productivity impact calculator: 1. Model determination. US Department of Agriculture. 1990.

[57] Sharp R, Chaplin-Kramer R, Wood S, Guerry A, Douglass J. InVEST User’s Guide. 2018. 10.13140/RG.2.2.32693.78567.

[58] Allen R, Pereira L, Raes D, Smith M, Allen RG, Pereira LS, et al. Crop Evapotranspiration: Guidelines for Computing Crop Water Requirements, FAO Irrigation and Drainage Paper 56. 1998;56.

[59] Zhang L, Hickel K, Dawes WR, Chiew FHS, Western AW, Briggs PR. A rational function approach for estimating mean annual evapotranspiration. Water Resources Research. 2004;40. (2). 10.1029/2003WR002710.

[60] Brunsdon C, Fotheringham AS, Charlton ME. Geographically Weighted Regression: A Method for Exploring Spatial Nonstationarity. 1996;28(4):281–98. 10.1111/j.1538-4632.1996.tb00936.x.

[61] Clement F, Orange D, Williams M, Mulley C, Epprecht M. Drivers of afforestation in Northern Vietnam: Assessing local variations using geographically weighted regression. Applied Geography. 2009;29:561–76. 10.1016/j.apgeog.2009.01.003.

[62] Foody GM. Geographical weighting as a further refinement to regression modelling: An example focused on the NDVI–rainfall relationship. Remote Sensing of Environment. 2003;88(3):283–93. 10.1016/j.rse.2003.08.004.

[63] Fotheringham A, Brunsdon C, Charlton M. Geographically Weighted Regression: The Analysis of Spatially Varying Relationships. John Wiley & Sons. 2002;13.

[64] Su S, Xiao R, Zhang Y. Multi-scale analysis of spatially varying relationships between agricultural landscape patterns and urbanization using geographically weighted regression. Applied Geography. 2012;32(2):360–75. 10.1016/j.apgeog.2011.06.005.

[65] Pineda Jaimes NB, Bosque Sendra J, Gómez Delgado M, Franco Plata R. Exploring the driving forces behind deforestation in the state of Mexico (Mexico) using geographically weighted regression. Applied Geography. 2010;30(4):576–91. 10.1016/j.apgeog.2010.05.004.

[66] Su S, Li D, Hu Yn, Xiao R, Zhang Y. Spatially non-stationary response of ecosystem service value changes to urbanization in Shanghai, China. Ecological Indicators. 2014;45:332–9. 10.1016/j.ecolind.2014.04.031.

[67] Sun Y, Guo Q, Liu J, Wang R. Scale Effects on Spatially Varying Relationships Between Urban Landscape Patterns and Water Quality. Environmental Management. 2014;54(2):272–87. doi: 10.1007/s00267-014-0287-x 24838413

[68] Li Zhao. Investigating the Spatiotemporally Varying Correlation between Urban Spatial Patterns and Ecosystem Services: A Case Study of Nansihu Lake Basin, China. ISPRS International Journal of Geo-Information. 2019;8. (8). 10.3390/ijgi8080346.

[69] Nakaya T, Fotheringham S, Charlton M, Brunsdon C, editors. Semiparametric geographically weighted generalised linear modelling in GWR 4.0. Geocomputation; 2009.

[70] Peng W, Zhou J, Yang C, Zhao J, Luo H. Research on ecosystem service values based on land use change in Sichuan Province. Resour Environ Yangtze Basin. 2014;23:1053–62 (in Chinese).

[71] Härdle WK, Simar L. Canonical Correlation Analysis. In: Härdle WK, Simar L, editors. Applied Multivariate Statistical Analysis. Cham: Springer International Publishing; 2019. p. 431–42.

[72] Borga M. Canonical Correlation a Tutorial. 2022.

[73] Tao T, Abades S, Teng S, Huang ZYX, Reino L, Chen BJW, et al. Macroecological factors shape local-scale spatial patterns in agriculturalist settlements. Proc Biol Sci. 2017;284(1866):20172003. doi: 10.1098/rspb.2017.2003 29118138 PMC5698655

[74] Row JR, Doherty KE, Cross TB, Schwartz MK, Oyler-McCance SJ, Naugle DE, et al. Quantifying functional connectivity: The role of breeding habitat, abundance, and landscape features on range-wide gene flow in sage-grouse. Evol Appl. 2018;11(8):1305–21. doi: 10.1111/eva.12627 30151042 PMC6099827

[75] Odgaard MV, Bøcher PK, Dalgaard T, Moeslund JE, Svenning J-C. Human-driven topographic effects on the distribution of forest in a flat, lowland agricultural region. Journal of Geographical Sciences. 2014;24(1):76–92. 10.1007/s11442-014-1074-6.

[76] Arabameri A, Blaschke T, Pradhan B, Pourghasemi HR, Tiefenbacher JP, Bui DT. Evaluation of Recent Advanced Soft Computing Techniques for Gully Erosion Susceptibility Mapping: A Comparative Study. Sensors (Basel). 2020;20(2):335. doi: 10.3390/s20020335 31936038 PMC7014250

[77] Liu Z, Wimberly MC. Climatic and Landscape Influences on Fire Regimes from 1984 to 2010 in the Western United States. PLoS One. 2015;10(10):e0140839–e. doi: 10.1371/journal.pone.0140839 26465959 PMC4605733

[78] Tzeng H-Y, Wang W, Tseng Y-H, Chiu C-A, Kuo C-C, Tsai S-T. Tree mortality in response to typhoon-induced floods and mudslides is determined by tree species, size, and position in a riparian Formosan gum forest in subtropical Taiwan. PLoS One. 2018;13(1):e0190832–e. doi: 10.1371/journal.pone.0190832 29304149 PMC5755898

[79] Sun Q, Wang R, Hu Y, Yao L, Guo S. Spatial variations of soil respiration and temperature sensitivity along a steep slope of the semiarid Loess Plateau. PLoS One. 2018;13(4):e0195400–e. doi: 10.1371/journal.pone.0195400 29624600 PMC5889173

[80] Zhang J, Wang T, Ge J. Assessing Vegetation Cover Dynamics Induced by Policy-Driven Ecological Restoration and Implication to Soil Erosion in Southern China. PLoS One. 2015;10(6):e0131352–e. doi: 10.1371/journal.pone.0131352 26115116 PMC4482633

[81] Andrew J., Guswa Kate, Brauman A., et al. Ecosystem services: Challenges and opportunities for hydrologic modeling to support decision making. Water Resources Research. 2014;50(5):4535–44. 10.1002/2014wr015497.

[82] Fu B, Xu P, Wang Y, Guo Y. Integrating Ecosystem Services and Human Demand for a New Ecosystem Management Approach: A Case Study from the Giant Panda World Heritage Site. Sustainability. 2019;12:295. 10.3390/su12010295.

[83] Han R, Sun SQ, Guo L, Chen YZ. Evolution of Ecosystem Service Value and Analysis of Driving Forces in the East Region of Sichuan Province, China. Journal of Ecology and Rural Environment (in Chinese). 2019.

